# Supplementary material for: Benefits of jasmonate-dependent defenses against vertebrate herbivores in nature
Source: eLife. 2016 Jun 29;5:e13720. doi: 10.7554/eLife.13720 (PMC4927296; doi:10.7554/eLife.13720)
Supplement: Figure 1—source data 1. — DOI: http://dx.doi.org/10.7554/eLife.13720.004 [file elife-13720-fig1-data1.pptx]

## Slide 1
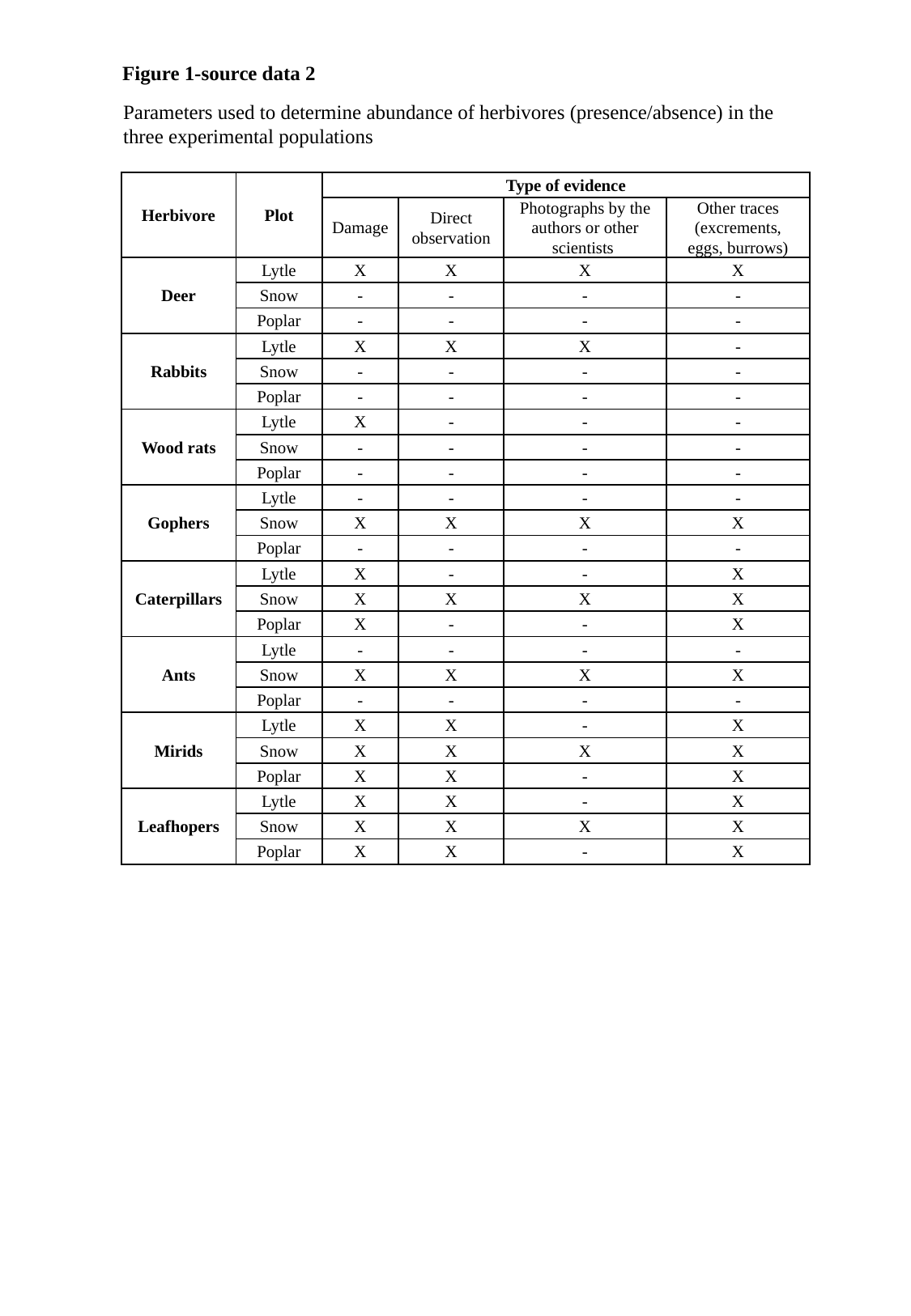

Figure 1-source data 2
Parameters used to determine abundance of herbivores (presence/absence) in the three experimental populations
| Herbivore | Plot | Type of evidence | | | |
| --- | --- | --- | --- | --- | --- |
| | | Damage | Direct observation | Photographs by the authors or other scientists | Other traces (excrements, eggs, burrows) |
| Deer | Lytle | X | X | X | X |
| | Snow | - | - | - | - |
| | Poplar | - | - | - | - |
| Rabbits | Lytle | X | X | X | - |
| | Snow | - | - | - | - |
| | Poplar | - | - | - | - |
| Wood rats | Lytle | X | - | - | - |
| | Snow | - | - | - | - |
| | Poplar | - | - | - | - |
| Gophers | Lytle | - | - | - | - |
| | Snow | X | X | X | X |
| | Poplar | - | - | - | - |
| Caterpillars | Lytle | X | - | - | X |
| | Snow | X | X | X | X |
| | Poplar | X | - | - | X |
| Ants | Lytle | - | - | - | - |
| | Snow | X | X | X | X |
| | Poplar | - | - | - | - |
| Mirids | Lytle | X | X | - | X |
| | Snow | X | X | X | X |
| | Poplar | X | X | - | X |
| Leafhopers | Lytle | X | X | - | X |
| | Snow | X | X | X | X |
| | Poplar | X | X | - | X |
