# Supplementary material for: Benefits of jasmonate-dependent defenses against vertebrate herbivores in nature
Source: eLife. 2016 Jun 29;5:e13720. doi: 10.7554/eLife.13720 (PMC4927296; doi:10.7554/eLife.13720)
Supplement: Figure 1—source data 2. — DOI: http://dx.doi.org/10.7554/eLife.13720.005 [file elife-13720-fig1-data2.pptx]

## Slide 1
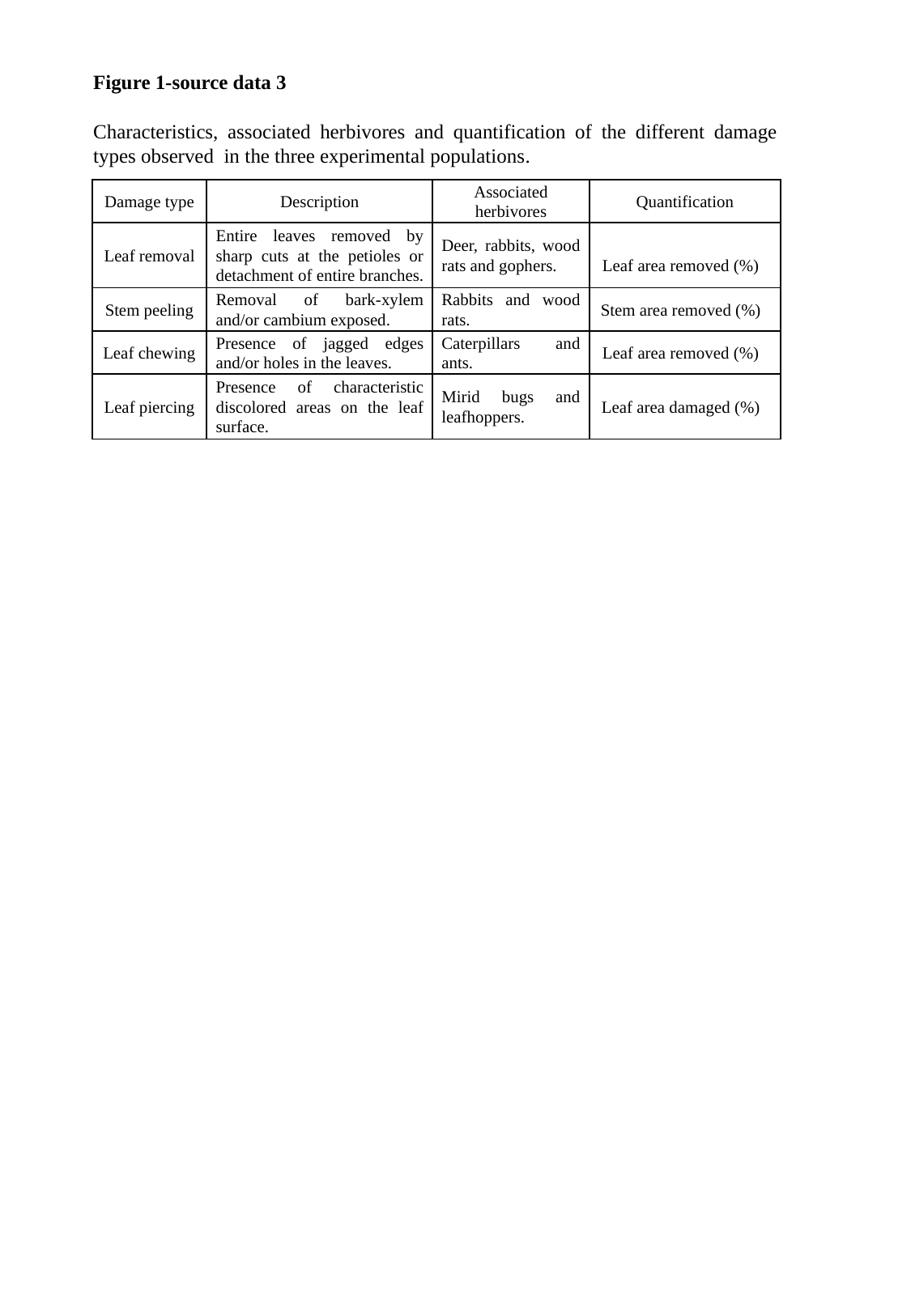

Figure 1-source data 3
Characteristics, associated herbivores and quantification of the different damage types observed in the three experimental populations.
| Damage type | Description | Associated herbivores | Quantification |
| --- | --- | --- | --- |
| Leaf removal | Entire leaves removed by sharp cuts at the petioles or detachment of entire branches. | Deer, rabbits, wood rats and gophers. | Leaf area removed (%) |
| Stem peeling | Removal of bark-xylem and/or cambium exposed. | Rabbits and wood rats. | Stem area removed (%) |
| Leaf chewing | Presence of jagged edges and/or holes in the leaves. | Caterpillars and ants. | Leaf area removed (%) |
| Leaf piercing | Presence of characteristic discolored areas on the leaf surface. | Mirid bugs and leafhoppers. | Leaf area damaged (%) |
